# Density Visualization Pipeline: A Tool for Cellular and Network Density Visualization and Analysis

**DOI:** 10.3389/fncom.2020.00042

**Published:** 2020-06-26

**Authors:** Stephan Grein, Guanxiao Qi, Gillian Queisser

**Affiliations:** ^1^Department of Mathematics, Temple University, Philadelphia, PA, United States; ^2^Institute of Neuroscience and Medicine (INM-10), Research Centre Jülich, Jülich, Germany

**Keywords:** GABAergic, barrel cortex, density visualization, visual programming, neuronal morphology, synaptogenesis, density maps, interactive data analysis

## Abstract

Neuron classification is an important component in analyzing network structure and quantifying the effect of neuron topology on signal processing. Current quantification and classification approaches rely on morphology projection onto lower-dimensional spaces. In this paper a 3D visualization and quantification tool is presented. The Density Visualization Pipeline (DVP) computes, visualizes and quantifies the density distribution, i.e., the “mass” of interneurons. We use the DVP to characterize and classify a set of GABAergic interneurons. Classification of GABAergic interneurons is of crucial importance to understand on the one hand their various functions and on the other hand their ubiquitous appearance in the neocortex. 3D density map visualization and projection to the one-dimensional x, y, z subspaces show a clear distinction between the studied cells, based on these metrics. The DVP can be coupled to computational studies of the behavior of neurons and networks, in which network topology information is derived from DVP information. The DVP reads common neuromorphological file formats, e.g., Neurolucida XML files, *NeuroMorpho.org* SWC files and plain ASCII files. Full 3D visualization and projections of the density to 1D and 2D manifolds are supported by the DVP. All routines are embedded within the visual programming IDE VRL-Studio for Java which allows the definition and rapid modification of analysis workflows.

## 1. Introduction

The stunning diversity of neuronal morphologies is being studied since the work of Cajal (Ramón y Cajal, 1899) a century ago. Since then, neuronal morphology has been considered an important component when analyzing rapidly growing neuroanatomical data. Neuronal morphology may be consulted to support or assist in characterization of neuronal function in local cortical microcircuits. Detailed reconstructions enable *in silico* experimentation of the electrical and chemical signals associated with specific cell types to study and validate structure-function interaction. The somato-dendritic and axonal morphologies reflect the neuronal input and output patterns: The former indicates the number and location of synaptic inputs while the latter defines the spatial distribution of synaptic outputs. When the axonal morphology of one neuron and the dendritic morphology of another neuron are both available, the spatial overlap between the axonal and dendritic trees can be used to calculate the location of potential synapses (Lubke et al., 2003; Stepanyants and Chklovskii, 2005; Jefferis et al., 2007; Helmstaedter et al., 2008; Levy and Reyes, 2012; Packer et al., 2013) motivated by Peters' rule (Peters and Feldman, 1976; Peters and Payne, 1983; Braitenberg and Schüz, 1991). With the development of neuronal reconstruction techniques [e.g., the Neurolucida system (MicroBrightField) (Aguiar et al., 2013) and (Halavi et al., 2012)] and several large-scale brain research projects and novel reconstruction methods (Peng et al., 2010, 2014; Bria et al., 2016), digital neuronal morphologies have been systematically acquired and became freely accessible, via projects and databases, such as *NeuroMorpho.org* (Ascoli et al., 2007), The Blue Brain Project (Markram et al., 2015), Allen Cell Types Database^1^, and Allen Brain Atlas (Sunkin et al., 2012; Gouwens et al., 2019).

Density maps are important for cell classification and judging where synaptic contacts could potentially be formed. Density maps are typically one- or two-dimensional projections of the neuronal morphology onto the x-, y-, or z-axis or to xy-, xz-, yz-plane. A density map can be thought of as a population density map, subdividing the x-, y-, or z-axis into 1D bins and respectively subdividing the planes into 2D bins for which one can calculate the neuronal morphology mass contained in the bin. For each bin the total neuronal length is normalized to yield a density in the range [0, 1]. For the purpose of neuronal visualization and morphometric analysis for the ever-growing data, the development of open source, highly efficient, multi-functional software becomes a necessary objective.

While morphometric analysis tools exist, there are some drawbacks that are addressed in the presented work. Commercial tools, such as the Neurolucida Explorer (MicroBrightField) allow for 1D and 2D density field visualization and analysis, but not a full three-dimensional method. Their source-code is proprietary and thus not extendible by the research community. Other toolboxes support 3D density analysis, like the TREES toolbox (Cuntz et al., 2010) or the Filament Editor (Dercksen et al., 2014), but rely on third-party commercial software, such as Matlab and Amira. Finally, freely available tools, like Py3DN (Aguiar et al., 2013) support certain analysis features, but are challenging to use and extend given their dependency on various plugins.

**Figure 1:**
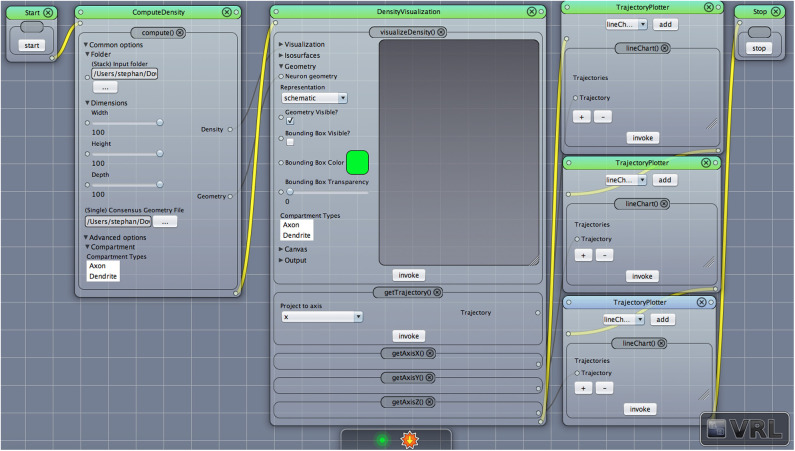
A VRL template project loaded from the included template projects folder in the plugin. The simulation workflow (data- and control-flow logic) is displayed. There are three main components, ComputeDensity component, the Densityvisualization component as well as the TrajectoryPlotter components which will plot the projected data to the x-, y-, and z-axis, respectively. Start and Stop components are available to start or stop the workflow.

Given some of the existing limitations, the presented Density Visualization Pipeline (DVP) has the following developmental objectives. The DVP framework should be self-contained, i.e., a single project file contains all dependencies and workflow information, and can be launched in VRL-Studio (Hoffer et al., 2013). Since data needs to be processed efficiently, either interactively or remotely, workflows need to be established to batch-process a large number of neuroanatomical data, thus, allow for a flexible workflow design via a declarative GUI programming framework. Leveraging parallel processing power enables processing large data sets. Furthermore, data should be visualized efficiently and analyzed by the same token and the workflow of visualization and analysis needs to be easily shared. Solving these challenges by employing a platform independent approach using the Java and Groovy programming language allows for flexible application and distribution of the analysis and visualization workflows (see Figure 1). Using the DVP in a computational research setting the functional objectives of the DVP include the ability to generate and visualize three-dimensional density maps (as well as projected 1D profiles), differentiate between dendritic and axonal density maps, visualize the unprojected neuronal morphology and cortical landmarks (layer borders and barrel borders in case of the barrel cortex and differs for the corpus of cortical reconstructions) and to export all generated data. The DVP and its' functional objectives are presented in sections 2.1–2.4.

Density maps have been applied to classify and characterize neuronal cell types in different cortical areas and species (Jefferis et al., 2007; Cuntz et al., 2008, 2012; Oberlaender et al., 2011; Sümbül et al., 2014a,b; Koelbl et al., 2015; Emmenegger et al., 2018) and to produce morphometric statistics which include detailed representation of the neuronal morphologies (Uylings and van Pelt, 2002; Scorcioni et al., 2008; Lu et al., 2013; Budd et al., 2015). Novel approaches worth mentioning are feature representation by graph theory and topology developed recently by Li et al. (2007), Heumann and Wittum (2009), Gillette and Grefenstette (2009), Gillette and Ascoli (2015), and Kanari et al. (2018, 2019). Thus, we here apply the DVP to a data set of neurons and demonstrate the ability to classify neuron types by their distinct density maps. The results are presented in sections 2.5–2.6. Such results can then be integrated in larger computational workflows, e.g., numerical simulation of electrical signal processing in neurons and networks (Breit et al., 2016; Stepniewski et al., 2019).

The DVP front-end is graphically controllable and completely separate from the programmable backend. Thus, the DVP is easy to use by neuroscience researchers. The presented framework nonetheless allows developers to seamlessly add new features to the backend and make them easily available to front-end users. This strategy is described in section 3.

## 2. Results

To judge where the mass of neurons is located and thus synapses may be formed (Peters and Feldman, 1976; Braitenberg and Schüz, 1991) density maps, as discussed by Peters and Payne(1983) can be a valuable tool. Such density maps enable the researcher to locate the spatial overlap of cells or functional parts of a cell, e.g., soma, axon, dendrite by proximity analysis and have been applied successfully to reveal design principles of dendritic arbors (Cuntz et al., 2008, 2012). Cell types can be furthermore discriminated by analyzing density maps and classes of cells might be defined by the characteristics of such density profiles (Sümbül et al., 2014b). Statistics arising from the density data can then be further used to classify and categorize the prevalent cells in e.g., different layers of some designated brain region or animal species (Oberlaender et al., 2011). Typically 1D and 2D density maps have been employed for such neuroanatomical analysis, however also 3D density maps (without projections to 1D or 2D manifolds) can potentially reveal characteristics of the cell morphologies which might be lost during projection. Due to the steadily increasing availability of neuroanatomical data, through novel reconstruction techniques and large-scale brain research projects like the Allen Brain Atlas (Sunkin et al., 2012), and stored in publicly available databases like NeuroMorpho.org (Ascoli et al., 2007) it is inevitable to devise an automatic, yet user-controllable, and thus interactive pipeline to analyze large data sets, potentially also in non-interactive batch mode for very large data sets. Classification of overlap regions are of crucial interest in the context of synapse loss and synaptogenesis and thus are of utmost importance for understanding neurodegenerative or learning processes. The DVP plugin addresses the automatization of data import, visualization and subsequent analysis of the projected and unprojected density data originating from neuroanatomical data.

### 2.1. Density Analysis and Customization of Data and Control-Flow Logic

In the following the main workflow will be described, in particular how to apply the DVP to given input data. For an overview of the workflow cf. Figure 1 which illustrates the main components in the workflow. After downloading, installing and opening VRL-Studio^2^ (Hoffer et al., 2013), the user can open the DVP template project which lays out all the components shown in Figure 1. User-modified templates can be saved to a VRLP file which is internally versioned with the distributed versioning framework Git in the VRLP project file itself. It is worth mentioning that the user can use the graphical UI of the DVP without any knowledge of the backend. The Java backend, which is described in section 3, can be accessed for adding new user-defined features and behaviours.

After opening the VRLP template project, the user can provide a folder of neuroanatomical input data which should be analyzed through the folder option in the DensityVisualization component. Additionally a single consensus geometry file can be specified which will contain the allowed compartments for visualization and density calculation. The consensus geometry file can also be used later to align all cells from the input folder with respect to the single consensus geometry file, e.g., center at soma. The consensus geometry file typically also provides contours for layer boundaries which makes it easier to identify specific regions in e.g., the Barrel Cortex and so on, see Figure 1 second column. Data connection lines between the components are colored in black color and the control-flow logic is defined by yellow lines. By default, the DensityVisualization component will visualize the density in 3D and all neuroanatomical data, i.e., cells and if present, layer boundaries which are provided by the single consensus geometry file. The currently supported file formats are listed in Table 1. However, custom importers can be swiftly added by the user. Next, the density profiles can be exported by a right-click mouse operation on the main canvas of the aforementioned component and per default the projections to the x-, y- and z-axes of the 3D density data is displayed in the right-most TrajectoryPlotter component. By clicking the Start component button the processing pipeline will be invoked and can be stopped prematurely if needed by the Stop component. All components have a variety of options to control the analysis and are summarized in Tables 2, 3.

**Table 1:** Supported file formats in the current version of the DVP plugin.

**Type**	**Description**	**Reference**	**Note**
SWC	Stores trees as connected cylindrical segments as specification of neural morphology (used in compartment neuronal modeling). The conversion from commercial, feature-rich formats to SWC comes with a loss of information. For example all lines, markers or contours that mark features of the neuron and its position in the tissue are lost.	Cannon et al. (1998)	SWC are the initials of the last names of E.W. Stockley, H.V. Wheal, and H.M. Cole, who developed a system for generating morphometric reconstructions of neurons that is described in Stockley et al. (1993).
XML	Hierarchical data format containing the modeling data for Neurolucida (dendrites, axons, somata, markers, spines) and for Stereo Investigator (probes, markers).	**https://www.mbfbioscience.com/help/si11/Content/About/FileFormats.htm**	XML (extensible markup language) version of the ASC modeling data file. This format contains some extensions for 3D mesh modeling not stored in the ASC and DAT file versions.
ASC	Non-hierarchical data format containing the modeling data for Neurolucida (dendrites, axons, somata, markers, spines) and for Stereo Investigator (probes, markers).	**https://www.mbfbioscience.com/help/si11/Content/About/FileFormats.htm**	Text version of the DAT modeling data file.
DAT	Non-hierarchical data format containing the modeling data for Neurolucida (dendrites, axons, somata, markers, spines) and for Stereo Investigator (probes, markers).	**https://www.mbfbioscience.com/help/si11/Content/About/FileFormats.htm**	*Not yet used or supported*

*Most neuroanatomical data is either specified in SWC, XML, or ASC respectively DAT file format (Neurolucida plain ASCII files) and thus these formats are primarily supported by the DVP plugin. However, additional file formats or types can be easily added by following the described procedure in the main text*.

**Table 2:** ComputeDensity parameters, types, allowed values, and description of intended use in the DVP plugin for VRL-Studio.

	**Type**	**Description**	**Range**	**Default**
**FOLDER**				
Input folder	Directory string	Specify the input folder for the neuroanatomical data	–	–
**DIMENSIONS**				
Width	Float	Width of sampling cuboid used during density calculation	[0, 100]	50
Height	Float	Height of sampling cuboid used during density calculation	[0, 100]	50
Depth	Float	Depth of sampling cuboid used during density calculation	[0, 100]	50
Consensus Geometry	File string	Specify the consensus geometry to align files from stack input folder	–	–
**COMPARTMENTS**				
Compartment types	List of strings	Specify the compartment types which should be ignored in density calculation. Compartment names from consensus geometry file	–	–

**Table 3:** DensityVisualization parameters, types, allowed values, and description of intended use in the DVP plugin for VRL-Studio.

	**Type**	**Description**	**Range**	**Default**
**VISUALIZATION**				
Min. Density	Integer	Threshold. Visualize all densities above this value.	[%]	0
Density Color 0	RGB color	Color representing 0 % density	RGB value	Blue
Density Color 1	RGB color	Color representing 100 % density	RGB value	Red
Density Transparency	Float	Enables density transparency for visualization [%]	[0, 1]	0
Density Visible?	Boolean	Enables or disables visualization of the density	–	True
**ISOSURFACES**				
Visible?	Boolean	Enables or disables visualization of isocontours	–	False
Average	Integer	Value of isocontours to be visualized	[%]	0
Deviation	Integer	Amount of deviation from average still to visualize	[%]	0
**GEOMETRY**				
Compartment Types	List of strings	Specifies which compartments of the geometry shall be	–	∅
		excluded from visualization		
**CANVAS**				
Scalebar visible?	Boolean	Add scalebar to canvas	–	True
Coordinate system visible?	Boolean	Add coordinate system to canvas	–	True
**OUTPUT**				
Blur	Matrix	Apply a blurring kernel to the density view	–	Gaussian blur
Animation	–	File type specified output format. Frames per second or seconds	Integer	0 and AVI format
		per frame can be specified		
Rotation	–	Rotation matrix can be specified to rotate density view in canvas		

### 2.2. Workflow Components

This section illustrates all plugin components and explains the available input and output options, parameter ranges, and meaning of the individual parameters which can be adjusted during interactive and iterative data analysis or batch-mode. Batch-mode projects can be created by setting up all of the control-flow and data-flow logic in the VRL-Studio GUI view or canvas and can be exported as a console application. Such a console application can be deployed to another computer which runs Java in headless mode to provide batch processing on a remote computing facility.

#### 2.2.1. The ComputeDensity Component

Entry point to the analysis is the ComputeDensity component displayed in Figure 1 (second column). User options are selection of an input folder specifying the location of user-acquired neuroanatomical data. The folder should contain the morphology files to be analyzed. The path is saved in a directory string for the DVP ComputeDensity component for subsequent processing or passing to other components of the DVP, see also the summarized options for this component in Table 2. Supported file types are listed in Table 1.

Each geometry file contains vertices v, edges ε which are defined by a pair of vertices, and diameter information associated with each vertex. To calculate a 3D density from the line-graph geometry, a sampling cuboid B with a user-specified size *l* is defined. The number *N*_*j*_ of sampling cuboids in x, y, and z direction (*j* = 1, 2, 3, respectively) is determined by

$$\begin{array}{l} N_{j}=\left\lceil \frac{E_{j}}{l}\right\rceil , \end{array}$$

where *E*_*j*_: = *U*_*j*_−*L*_*j*_ and

$$\begin{array}{l} L_{j}=\overset{\left|V\right|}{\underset{i=0}{\min}}V_{i_{j}},\;U_{j}=\overset{\left|V\right|}{\underset{i=0}{\max}}V_{i_{j}} \end{array}$$

are the minimum and maximum coordinate of all loaded cell geometries, respectively. The density |B| within a given B is determined by summing the length of all segments contained in B and then normalizing by the total segment length of a given neuron. When analyzing multiple neurons at once, this process is repeated for each neuron. The total density then is

$$\begin{array}{l} \left|B\right|=\frac{1}{m}\sum_{i=0}^{n}\left|B_{i}\right|, \end{array}$$

where *n* denotes the total number of neurons, |B_*i*_| the density of neuron *i* in a given sampling cuboid B, and $m=\max_{B}\left|B\right|$.

Having selected an input folder for density analysis the user needs to specify the granularity of the sampling. Since 3D data is available, the user can specify the dimension for the three coordinate axes. Typically the sampling is performed using a cuboid, where the x, y, and z dimension are positive integers. With the same value, e.g., *x* = *y* = *z* = 50μ*m*, i.e., the cuboid degenerates to a regular bounding or sampling cube. Before sampling the input neurons can be aligned to a point of reference with help of the consensus geometry file. The sampling of the density can exclude compartments of the input neurons, e.g., depending on the availability of the soma, axon or dendrite compartments in the neurons, one can exclude one or multiple compartments for density analysis. The ComputeDensity component has no inputs from other components since it is the starting point of the analysis pipeline, but forwards the Density and the Geometry data structures to the DensityVisualization component which uses these data structures to visualize the geometry and layer boundaries specified by a consensus geometry file and the ComputeDensity component actually calculates the density maps in 3D by using a parallel algorithm which subdivides the bounding box enclosing all neuronal compartments into a cuboid-like lattice by the number of available processors to accomplish a work sharing. The geometry is read in depending on the supported file type in an appropriate Geometry data structure, which is derived from a common Java interface, which is also used ultimately during the visualization of the neuronal morphologies by means of DensityVisualization component.

#### 2.2.2. The DensityVisualization Component

To visualize the density and neuronal geometry which was calculated and converted by the ComputeDensity component to an internal representation suitable. for the DensityVisualization component, see Figure 1 (third column), the data can be further analyzed. For an overview of options for the DensityVisualization component, see Table 3. The user can choose which density values to visualize. If all density values should be considered for visualization then the option *minimum density in percentage* can be left unchanged. The user can choose, by increasing or decreasing the density value, to visualize only voxels which lie above the chosen threshold value. Furthermore density colors for the zero density and for the unit density can be specified. In the visual representation these colors are mixed linearly to color code all density values. Density transparency can be toggled such that the neuronal morphologies can be made visible within the density voxels and are not occluded by the otherwise solid-colored density voxels. The options are summarized by the *Visualization tab* in the aforementioned component. The next adjustable group settings are the *isosurfaces options*. If the checkbox is marked then isosurfaces will be visualized on top of the geometry and density view. To calculate the isosurface in the DVP plugin a parallel implementation of the marching cubes algorithms was used to create contours of an average density in percentage and a deviation in percentage, see options for this settings group in Table 3. Threshold and deviation for visualization of these contours are adjustable. The *geometry* tab lets the user specify whether the neuronal morphologies should be visible or not by marking a checkbox. The spatial extent of all neuronal morphologies, e.g., bounding box or bounding cuboid, can be visualized if the corresponding checkbox is selected. The user can specify a bounding box color as well as a transparency value in percentage which allows the user to make all components visible and not occlude the view. One can exclude compartments from being considered during processing and display in the density visualization view. The user can choose between the representation to use for visualization of the neuronal geometries, i.e., either the neuronal geometries are approximated in the view as piecewise linear cylinders in 3D or by a line-graph structure, e.g., only edges and vertices are used. In the following “cylinder” refers to the first and “schematic” to the second representation type. The canvas allows to add a coordinate system and axes to the visualization as well as a scale bar for the neuronal morphologies to provide a measure to compare sizes of the cells automatically. The *output* tab summarizes the various output options. First, the resulting images can be blurred by a blurring kernel prior to saving the image to JPG, PNG, GIF, or TIFF. This option might be useful when saving the calculated voxel densities, which are discrete by nature. To create rotating animations of the view a rotation matrix might be specified by the user prior to exporting the AVI, MOV, or MPG video file by the *animation* tab parameters. To export statistics of the 1D profiles one can use the provided methods getAxisX(), getAxisY(), and getAxisZ() which export a general “trajectory” data structure or XYData to provide the line chart plotter (TrajectoryPlotter component) with data. Notice that by a right-click mouse operation on the main density visualization canvas, on the right hand side, a popup menu opens which allows to reset the view, save images, increase and decrease pan speed, increase and decrease translation increment and toggle a rotational view as well as toggle an animation and save the animation as a video. To save the blurred image a special save dialog is available to the user. Note that the user can use mouse chording operations in the presented view, e.g. zoom-in and zoom-out in the z-direction can be accomplished by using the mouse wheel, whereas left-click mouse down is used to rotate the view while moving the mouse.

#### 2.2.3. The TrajectoryPlotter Component

This component receives data input from the general XYData class provided by the component DensityVisualization to create line chart views. The default project setting is shown in Figure 1, (forth column), adds the three coordinate axes, x, y, and z and each coordinate axis is plotted in a distinct component view. It is also possible to combine all three line charts in one TrajectoryPlotter component. The TrajectoryPlotter component can save the plotted data by a save and export dialog to a variety of image formats. The available options are summarized in Table 4.

**Table 4:** TrajectoryPlotter parameters, types, allowed values, and description of intended use.

**Function name**	**Type**	**Description**	**Range**
lineChart()	Trajectory	Plots a specified trajectory	XYData
lineCharts()	Array of trajectories	Plots specified array of trajectory	XYData[]

*Note that the square brackets indicates an array data type*.

### 2.3. Data Import and Supported Formats

Typically neuroanatomical data is stored in a database like *NeuroMorpho.org* (Ascoli et al., 2007) as point-diameter data recorded in a plain text file of type SWC and line by line for each point and diameter. SWC files store the compartment type, e.g., axon, dendrite, soma by a numeric identifier, a running index, the 3D coordinates x, y and z as well as the diameter for each traced respectively recorded point and the connectivity information. The DVP allows the use of these traditional SWC files and is not necessarily restricted to this database or file type. SWC format represents the neuronal morphology by a list of nodes or points. Each item connects to its parent with a straight line by specifying an identifier. Additionally, ASCII files and XML files which have been exported by Neurolucida are supported. Hence, there is no need to use software, such as NLMorphologyConverter^3^ (Aguiar et al., 2013) to convert data and thus the DVP is self-contained. XML files are parsed by traversing the tree structure and are stored in a list of edges and vertices. If no header for the XML file is provided or the header is corrupted an auto-correction attempt of the XML file is performed. SWC files are stored in a similar manner. XML files record colors for the compartments whereas for SWC files one needs to assign either automatically or user-specified colors for the compartments. If more than one file is provided as input data for the DVP the data needs to be aligned by a point of reference, or, if using data exported by Neurolucida, this step can be omitted since Neurolucida usually aligns a stack at the soma. If other data formats are required, an interface, DensityVisualizable, is provided which can be used to implement a raw data import for an arbitrary custom file format which facilitates the data visualization and analysis. The density data is usually described by voxels but may be changed through the interfaces Density and Geometry. Data import and visualization can be performed in “batch-mode” which the user specifies a folder of interest and all neuronal morphologies are processed and visualized successively and added to the density and geometry view in VRL-Studio via batch processing.

### 2.4. Morphology and Density Visualization

**Figure 2:**
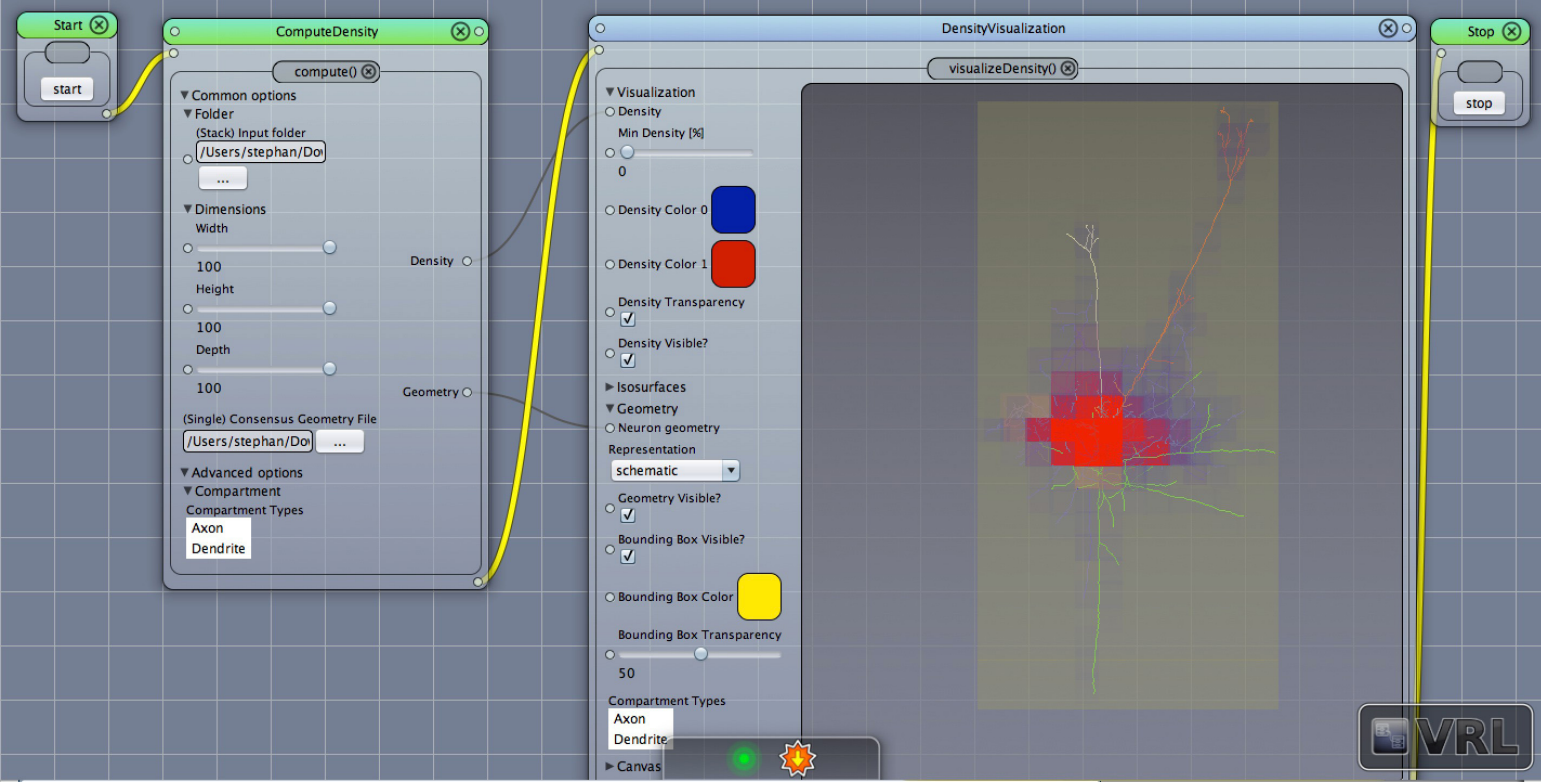
Density view. Displayed is the main Canvas view in VRL-Studio. Note that the workflow is organized from left to right, thus ComputeDensity is invoked first, then DensityVisualization. The graphical representations show a neuronal morphology with density voxels with specified size in the leftmost component and the neuron is represented as a line-graph consisting of vertices and edges. Note that the yellow bounding box indicates the spatial extent of all considered neuronal morphologies.

**Figure 3:**
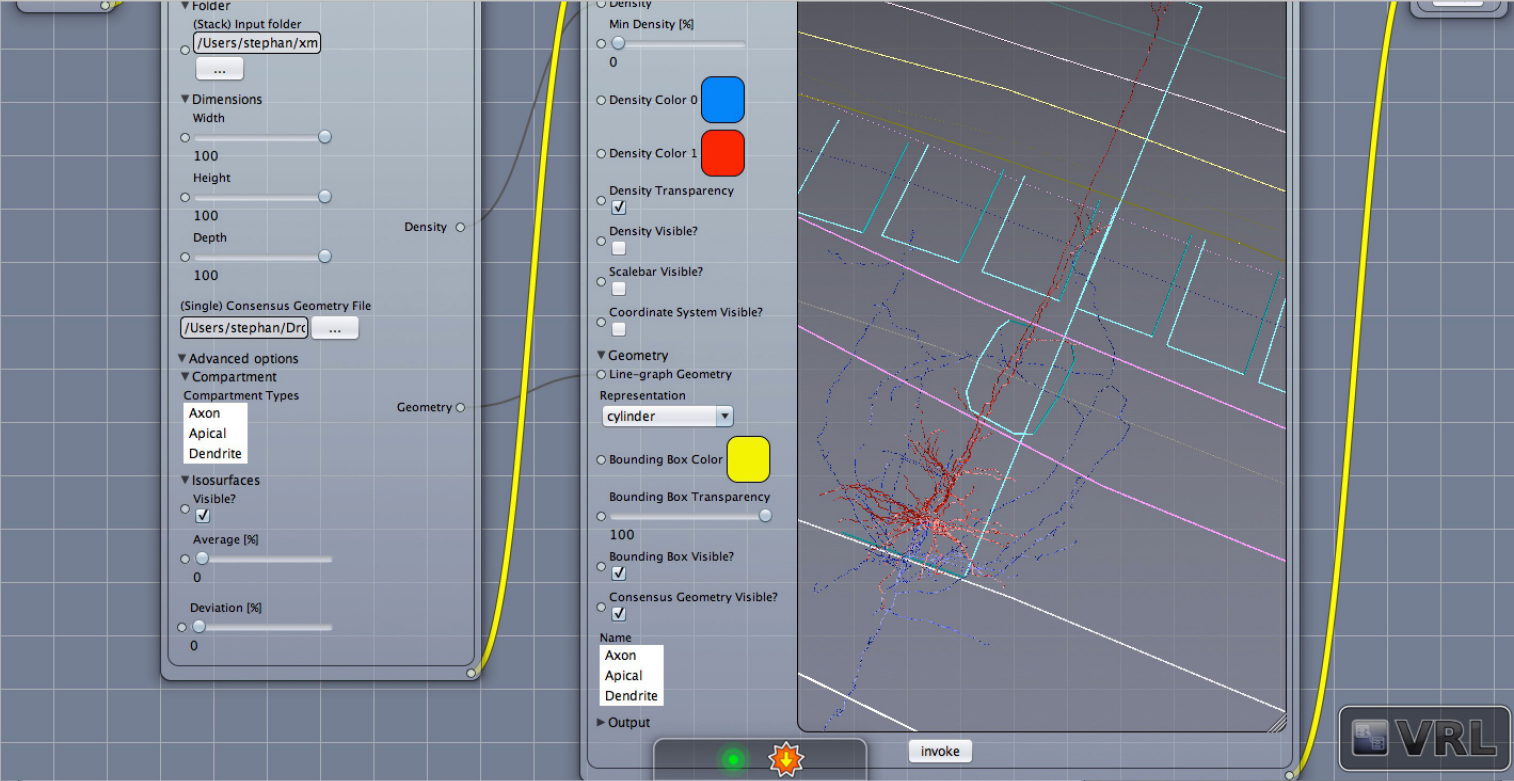
Geometry view with layer borders. Displayed is the main canvas view in VRL-Studio. A similar view as in Figure 2 is provided except the density is not visualized.

**Figure 4:**
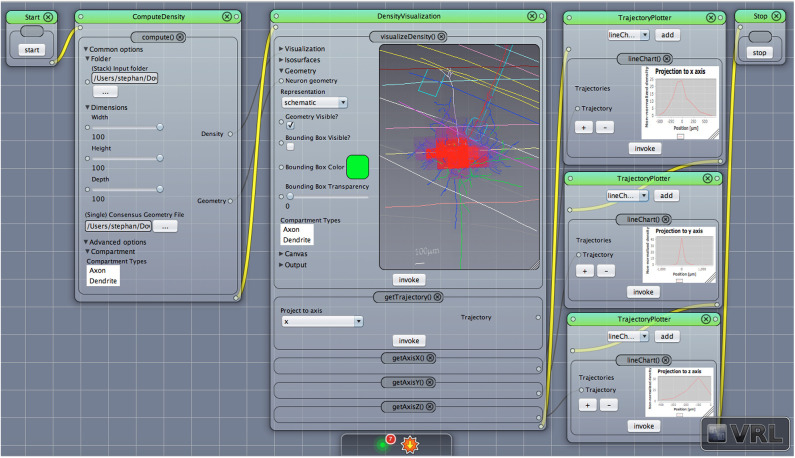
Density view with projection to axes. The default main canvas view in VRL-Studio is presented. Schematic representations of a pyramidal cell with layer boundaries are rendered and the density data (not shown) is projected to the x, y, and z-axis, respectively.

The DVP allows viewing the morphology and density in a 3D viewer embedded in VRL-Studio. The spatial structure can be inspected by rotation, zooming and translation of the camera using the mouse or keyboard. Compartments are color-coded according to the description in the data files and compartments can be excluded for analysis and visualization—or both—by a drop-down menu in the GUI. The neuronal morphology can either be displayed in 3D schematically as a line graph to only display the topological or connectivity structure of the cells of interest or by utilizing the diameter informations stored in the input data to create a cylinder graph representation. The latter representation uses *per se* more memory than the simple line graph representation and might not be advisable to use for fairly large neuronal morphologies. 3D densities (see Figure 2) and isocontours can be added to the view. To calculate densities one needs to specify a physiological length scale for the voxels, then the length of the neuronal morphologies are summed and normalized by the voxel volume to produce a density value for a given voxel. Voxel dimensions can be specified in pixels to control the level of detail of the density visualization. To add isosurfaces to the visualization, two parameters need to be specified, i.e., a mean value and a standard deviation. Isosurfaces are then generated by means of a parallel implementation of the marching cubes algorithm, see Lorensen and Cline (1987), and added to the visualization view in VRL-Studio (cf. Figure 2). The colormap of the density view can be adjusted as well and the isovalue can be specified for contours. The contours or cortical landmarks of the surrogate cell in the examples, e.g., Barrel border, can be visualized (see Figure 3 and Figure S2). Scale bar and coordinate axes are calculated automatically and can be added by the user. Density data can be projected to the x-, or- or z-axis (or to a user-specified plane or line) and can be plotted by using the ProjectToXYZAxisDensityDecorator (cf. Figure 4). In addition the user can write custom projections by implementing a decorator for the DensityVisualizable as mentioned before. Density profiles can be exported as images and rotational views can be exported as an animation from within the GUI, currently supported formats are PNG, GIF, and JPEG.

### 2.5. Neuromorphological Analysis

Neuromorphological analysis comprises both characterization (Costa and Velte, 1999; Costa et al., 2007) and classification (Bota and Swanson, 2007) of neuronal cells through multivariate techniques, which require choosing appropriate measurements (Costa, 1995) and the application of pattern recognition methods. A particularly relevant approach involves the grouping of neuronal cells into categories according to their morphological similarity. Such an approach is important for understanding the heterogeneity of the groups, as well as for unveiling the relationship between neuronal structure and function, and can be applied to comparative anatomy, developmental neurobiology, and diagnosis.

One of the most promising recent trends in neuroscience has been the advent of public data repositories, such as the *NeuroMorpho.org* database (Ascoli et al., 2007). Initiated in 2006, this database has grown steadily to become what is the most complete database of neuronal morphology, comprising currently^4^ a total of 112,244 cell reconstructions of several different types and species. It includes 3D reconstructions, measurements, software, and general information about the cells, such as reference papers, animal species, brain region, neuron class, amongst many others.

Typically there are three kinds of analyses one can conduct with neuroanatomical data at hand which can be extracted from such databases:

1. Visualization of neuronal morphology (soma, dendrites, and axons) and cortical landmarks (layer borders and barrel borders)
2. Calculation of the 3D density maps of dendritic and axonal branches and 1D profiles
3. Visualization of 3D (and 1D) density maps in addition to neuronal morphology

**Figure 5:**
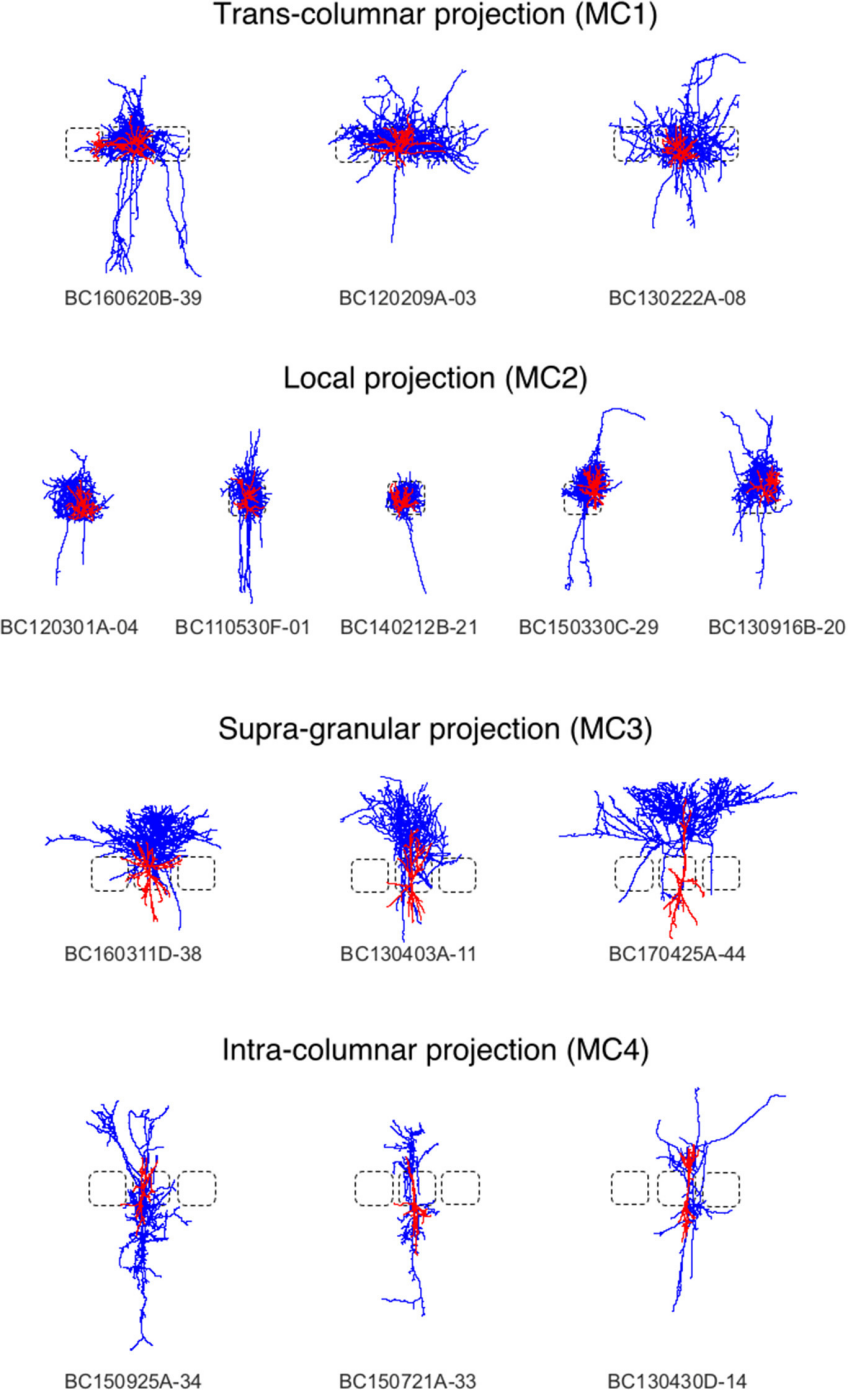
Interneurons of the rat barrel cortex which are composed out of neurons from the Trans-columnar projection (MC1), Local Projection (MC2), Supra-granular projection (MC3), and Intra-columnar projection (MC4).

Density maps are of interest for cell type discrimination (Oberlaender et al., 2011; Jiang et al., 2015) emphasized recently. To this end interneurons of the rat barrel cortex have been analyzed in the following paragraphs which are composed out of neurons from the Trans-columnar projection (MC1), Local Projection (MC2), Supra-granular projection (MC3), and Intra-columnar projection (MC4). The schematic neuronal morphologies and cortical landmarks can be found in Figure 5. The neuronal morphology can be visualized, which in this example is comprised out of soma, dendrites and axons compartments only (see Figure S1). A bounding box can be displayed to indicate the extent of the neuronal morphologies in the three dimensional space (not shown). Another possibility to visualize the data is to provide instead only the view of the neuronal morphology and in addition display the cortical landmarks of the barrel cortex as point of reference for navigation through the cell structure, scale bars and coordinate axes can be added through the graphical user interface (cf. Figure 3).

**Figure 6:**
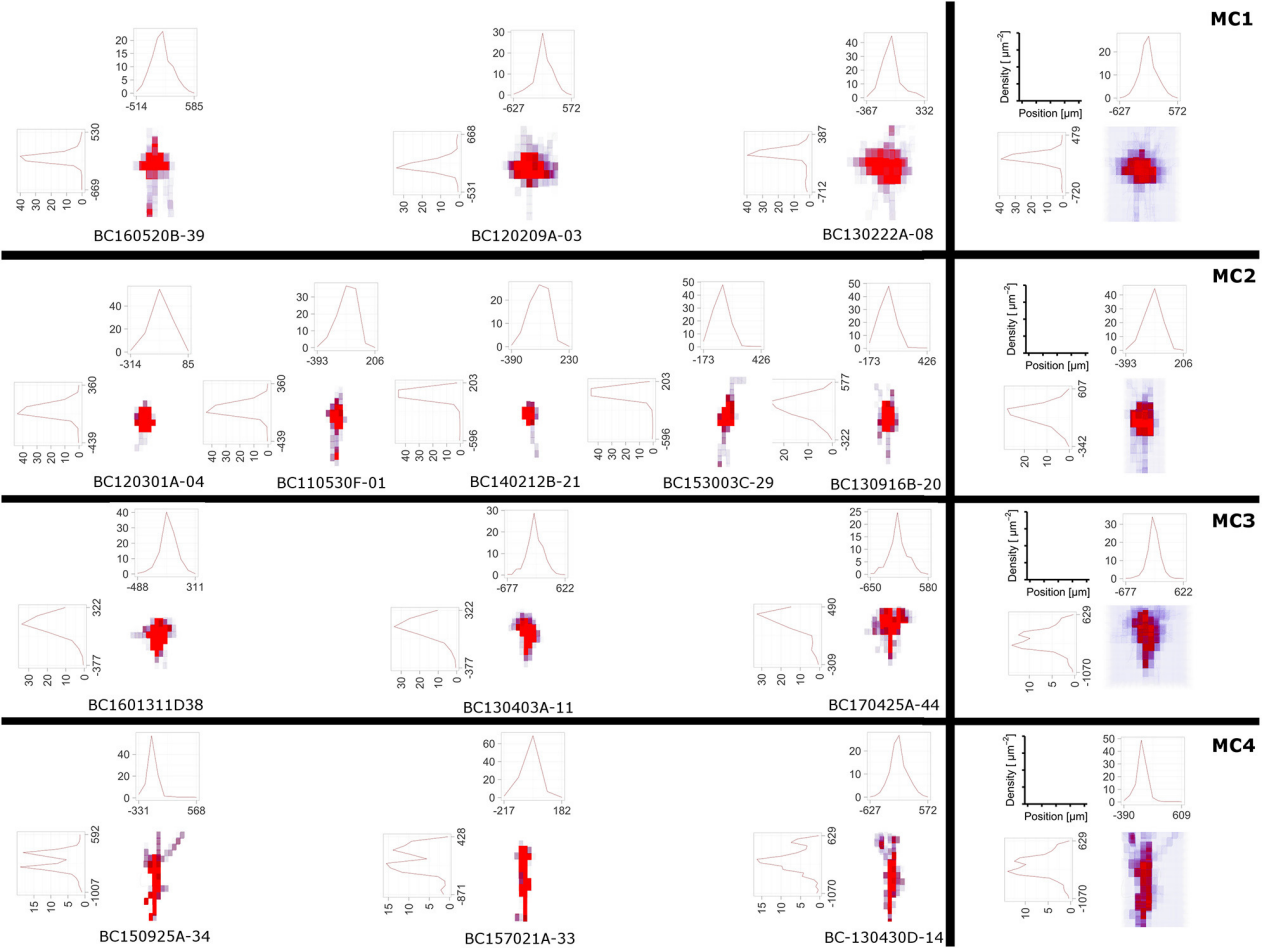
The DVP was applied to layer 4 non-fast spiking interneuron morphologies, where four morphological clusters (MCs) were obtained using the unsupervised cluster analysis based on their morphological features (Emmenegger et al., 2018). In addition to 3D density maps for individual interneurons within each MC **(left)**, the average 3D density maps for each MC **(right)** were calculated. Furthermore, the 1D profile of density maps in vertical and horizontal direction is given. From the density maps, it can be clearly seen that the 4 MCs show distinct patterns of dendritic and especially axonal arborization.

To judge where the “mass” of neurons is located one can employ density maps. To gain a better understanding of how the density is distributed a 3D density map might help. The 3D density maps of the interneurons can be found in Figure 6. As reported before the density maps might indicate where synapses can be formed in the brain. Another important analysis encompasses the calculation of density maps and plotting the corresponding 1D profiles, see Figure 4 for a projection of the density data to the three coordinate axes, e.g., x-, y-, and z-axis with the corresponding plots of the density distribution. Another task is to visualize the isocontours of the density data.

Density maps of lower dimensionality, e.g., 1D or 2D, are generated by projections of the neuronal morphology to a line or a plane. While the DVP plugin allows the specification of an arbitrary plane or line, it is customary to project data to either 1D lines which correspond to one of the coordinate axes or 2D planes which are a combination respectively pair of the coordinate axes, x-, y-, and z-axis, e.g., xy-plane, xz-plane, and yz-plane. The projected data and 1D density maps of the interneurons can be found in Figure 6 and evidently the profiles appear distinct. Lastly, a common theme in the analysis of neuroanatomical data is to overlay the 3D density maps with the cell morphologies themselves. This can be done as well, see Figure 2 and Figures S1, S2 and is useful when analyzing the overall structure and “hotspots” of synaptic density.

### 2.6. Characterization and Classification of Neocortical GABAergic Interneurons

Of the three commonly used methods, i.e., morphological, electrophysiological, and molecular, to characterize and classify cortical neurons the morphological method is regularly employed since it is relatively stable. The drawback to this approach however is that it is rather time-consuming (DeFelipe et al., 2013; Seung and Sumbul, 2014; Zeng and Sanes, 2017).

Morphologies of different cell types for characterization and classification, morphologies of dendritic and axonal structures have been obtained from stained and labeled neurons using a light microscopy-based reconstruction system for retina ganglion cells (Sümbül et al., 2014a) neocortical pyramidal cells (Oberlaender et al., 2011; Narayanan et al., 2015; Kanari et al., 2019; Egger et al., 2020) and neocortical GABAergic interneurons (Helmstaedter et al., 2008; Jiang et al., 2015; Koelbl et al., 2015) as well as (Emmenegger et al., 2018; Gouwens et al., 2019; Scala et al., 2019). In addition, neuronal morphologies could also be generated through a variety of computational modeling approaches (Ascoli et al., 2001) as well as (Cuntz et al., 2008, 2012; Wolf et al., 2013).

Here, we applied the DVP to the characterization and classification of non-fast spiking GABAergic interneurons in layer 4 of rat primary somatosensory (barrel) cortex. Four morphological clusters (MCs) are identified as: (1) Trans-columnar projection; (2) Local projection; (3) Supra-granular projection; (4) Intra-columnar projection have been obtained using the unsupervised hierarchical cluster analysis on dendritic and axonal parameters (Emmenegger et al., 2018). Representative examples for each of the MCs are displayed in Figure 5. The axonal and dendritic arborization pattern for neurons within the same cluster show a high similarity while for neurons from different clusters shows a prominent difference. To illustrate the general behavior of axonal and dendritic arborization pattern for neurons within each MC, length density maps of individual neurons and their average are generated and visualized in the xy-plane (Figure 6). In addition, 1D profiles of densities in vertical and horizontal directions (canonical coordinate axes) are also given. From the dendritic and axonal density maps, the potential input and output connectivities of neurons in each MC could be predicted according to the Peters' rule, which imply the specific functional role of each interneuron type in information processing within local microcircuits.

## 3. Materials and Methods

The Density Visualization Pipeline was implemented as a plugin for the integrated development environment VRL-Studio^5^, a declarative programming framework for the Java and Groovy programming language for the platforms Linux, OSX, and Windows. VRL-Studio provides a customizable graphical user interface (GUI), which allows development of workflows by visual programming, i.e., by manipulating and rearranging GUI elements and thus creating interactive program and data workflows. The workflows can be programmed by traditional programming paradigms (Java or Groovy), where textual representation and the code representation is visualized by the use of the visual reflection library (VRL^6^) established by Hoffer et al. (2013) and available in VRL-Studio. The user can switch between graphical code representation via a toggle in the GUI's main view and text-based code representation. The Java virtual machine (JVM) language Groovy is available from within VRL-Studio out of the box, which allows a rapid prototyping of work- and dataflow components. Unlike traditional programming which has not readily a visual representation of code available, visual programming is capable of representing textual code as graphical components immediately in a GUI view which allows for a more direct design of complex control-flow and data-flow logic aided by visual representation of the underlying code. As mentioned before VRL-Studio provides the possibility to decompile the graphical components via a toggle to reveal the underlying textual code representation. This is possible by using the Groovy language which runs on the JVM and by means of the VRL through code introspection. Through this mechanism the user is empowered to switch seemlessly to and between the appropriate representation(s) of the program depending on the user context and application, see Listing 1: Control-flow and data-flow logic, illustrating how a user can create a graphical component from Groovy or Java code and the resulting graphical representation is shown in Figure S3.

### 3.1. VRL-Studio Components

New components are introduced by the VRL annotation @ComponentInfo which specifies the name of a component as well as a category. The category is used to group multiple components, with the option of introducing multiple levels of grouping. Although Java code has advantages concerning runtime, the equivalent code can be defined within VRL-Studio by Groovy code basically (omitting the public modifier in the Listing 1). The advantage of defining components in Groovy is that one can decompile the graphical representation of the component into the code and *vice versa*. In this example the component is grouped under the group CustomGroup which in turn is subdivided in this example into one further subgroup Examples. Since Integers are built-in data types they can be represented graphically by the default representation of the type in VRL. For custom data types a custom type representation, which described how the data type should be rendered on the canvas, needs to be added.

**Listings 1:**
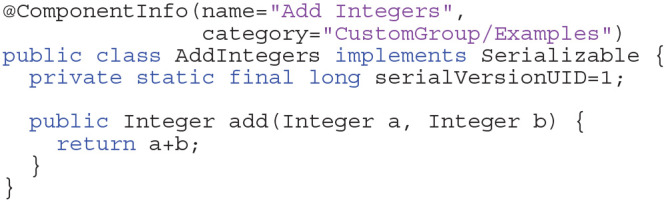
Example for adding two integers (Java).

**Figure 7:**
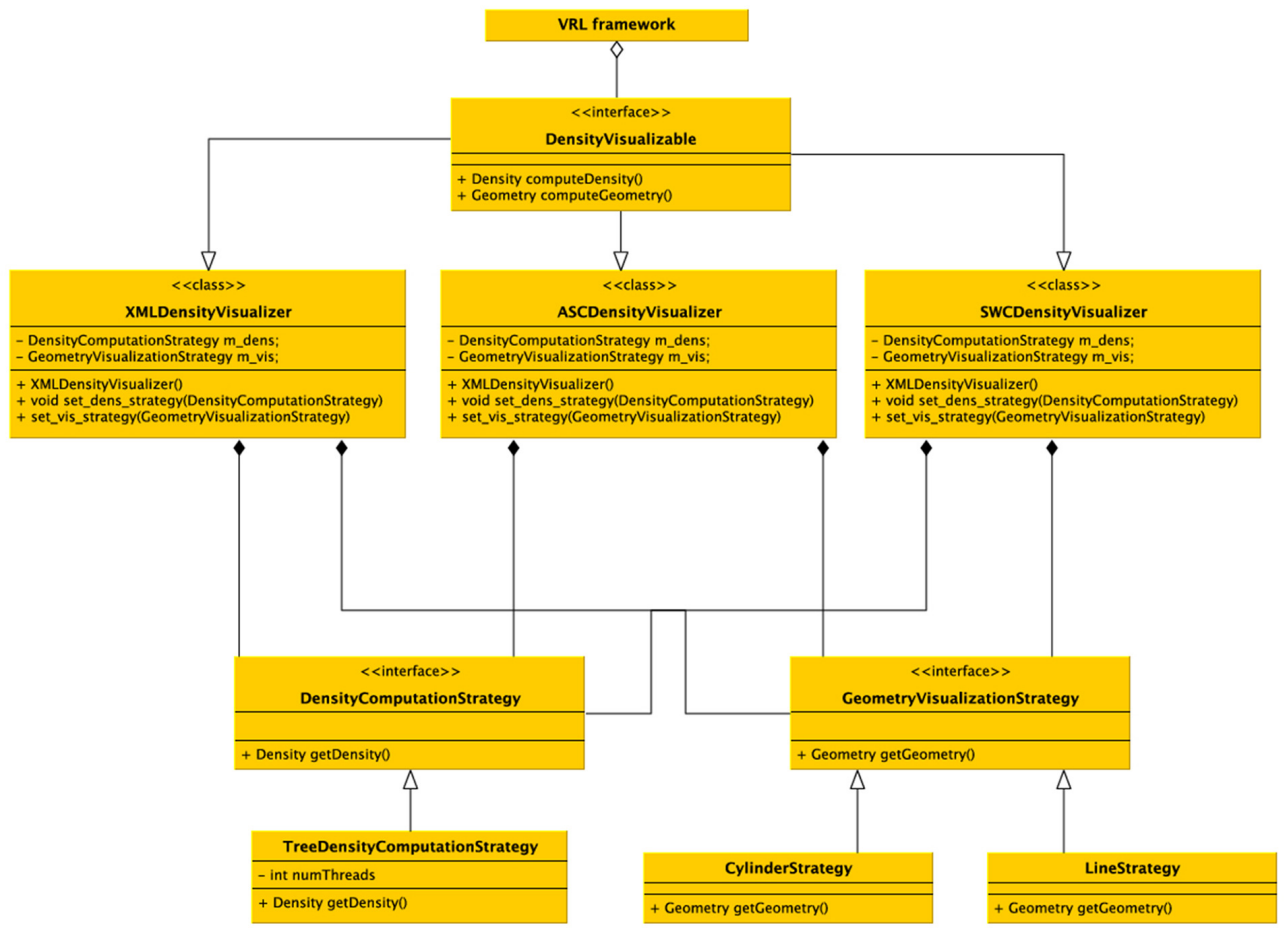
UML diagram of the density visualization pipeline. The class DensityVisualizable is aggregated in the VRL framework. For each (supported) file format a derived class is available which implements the format specific imports and density visualization. Different (generic) computational strategies exist, and for now the main strategy is to subdivide the data into a kd-tree and calculate densities in parallel depending on the number of processors available on the computer. Visualizing two generic strategies is possible, either a schematic representations by a line-graph geometry (edges and vertices) which are colored by the color specified in the data file or a cylindrical representation in 3D. Using the generic visualizers no additional implementation is required, but custom visualizers and density computors can be implemented easily by the provided interfaces, only the data format importer actually has to be implemented if not already supported by the DVP plugin.

VRL makes use of Java annotations which are a form of syntactic metadata that can be added to Java source code. The annotations can be retained by the JVM at runtime and read via reflection or introspection methods. This technique is used to create graphical representations of standard Java types or custom types which can be compiled to type representations. The DVP was designed as a plugin for VRL-Studio. A plugin defines an additional functionality which can be added to the VRL-Studio IDE at runtime by using the plugin mechanisms Add and Remove via the drop-down menu for Plugins in the VRL-Studio GUI. The scripting language Groovy for the JVM is also available for VRL-Studio and thus the designed DVP can be enhanced at runtime in the GUI editor of VRL-Studio. The plugins for VRL-Studio, and in particular the DVP, are self-contained, i.e., no additional runtime dependencies are required except VRL-Studio, which ships with an integrated Java runtime environment (JRE). The JRE is not hardwired into VRL-Studio and can be exchanged by the user if necessary. To extend plugins in VRL-Studio or the DVP the user needs in addition a Java development kit (JDK) to program in Java. Groovy is supported out-of-the-box through the VRL-Studio IDE. Each VRL-Studio plugin corresponds to a Java Netbeans respectively Java Gradle project and can be compiled to a single JAR file. The plugin can be either installed via VRL-Studio's GUI by using the plugins tab or can be copied to the VRL-Studio home directory .vrl/current in the user-specific directory. Since the plugin is a single JAR file, deployment to different platforms reduces to sharing one single file without platform-specific overhead and ceremony deployment and allowing to easily share the workflows used to carry out neuroanatomical data analysis. Each plugin can be accompanied by template projects which define recurring workflows (see Figure 1). These templates can be found in the drop-down menu File in the sub-menu Project from Template. Embedded in the template system is a Git versioning system which can be controlled via the VRL-Studio GUI, making it easy to roll back to old versions. Furthermore the necessity to include additional libraries can be satisfied by adding dependencies to the Java project and compile the project to a plugin. The software is distributed as a Gradle or Netbeans project or as a pre-compiled plugin packaged into a single JAR file or ZIP file licensed open-source (see section 5). To obtain VRL-Studio a direct link to the current version for OSX, Linux and Windows is provided on the project website and an archive of older versions for legacy projects is maintained on the website^7^. For further illustrations and an overview of the main workflow and the implementation details one may refer to Figures 1, 7.

### 3.2. Interactive Data-Analysis and Code Customization

The template project structure in Figure 1 provides control-flow logic, e.g., a Start and Stop component, and data-flow logic. Each graphical component (instance) in VRL-Studio can be recognized by a header with a unique name or identifier which corresponds to exactly one component class which is the code representation in either Groovy or Java code. For all built-in data types in Java a so-called type representation for the component can be defined, which overrides the default visual appearance of the corresponding component on the canvas, see Listing 2 for an excerpt of the Shape3dArrayTypeRepresentation which dictates the appearance of the neuronal morphology in the VisualizeDensity component. One can observe the TypeInfo annotation, which specifies the type, input and output behavior, and a style name for the class as a mnemonic for the user. All type representations need to inherit from the VRL type representation base class TypeRepresentationBase.

**Listings 2:**
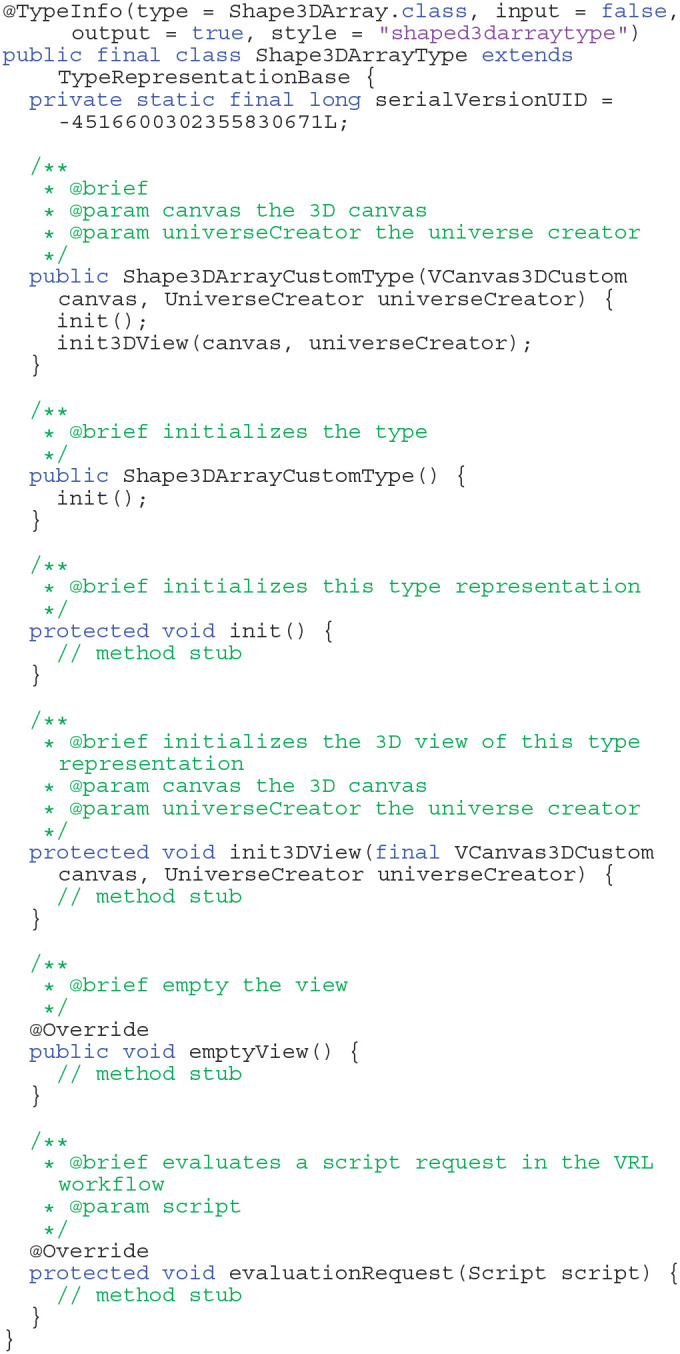
Example for a custom type representation for a custom type.

The VRL-studio canvas is represented as a grid and there are different color schemes available, which can be controlled through the View sub-menu. Components can be added to the canvas by the Components sub-menu which can be enabled by using right-click mouse operation. Observe that the control-flow corners logic is defined by connecting the white circles in the top left of each component with another component. The control-flow logic is visualized by the yellow curved lines connecting the component (see Figure 1). In addition, data can be shared between components as input or output. This fact is depicted in the template project by the black lines between the components. Using Java annotations the input and output names of members can be changed for the visual representation of the type which is dictated by the type representation for the built-in types or can be altered by custom type representations as explained earlier.

**Listings 3:**
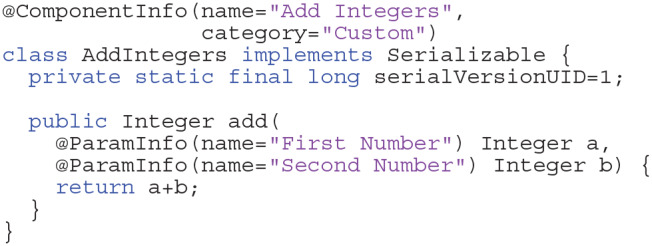
Example for adding two integers (Groovy).

Finally, it is worth mentioning that the DVP can be executed remotely in batch-mode. On a remote computing facility or a compute cluster the DVP enables users to process large number of cells on a remote resource, leveraging the processing power of parallel computing when subdividing the data sets in an appropriate fashion suitable for the compute resource of interest.

## 4. Discussion

This paper presents the Density Visualization Pipeline (DVP), a project that focusses on three-dimensional visualization and analysis of cellular mass distribution for cell classification. The DVP was implemented in Java using the VRL-Studio framework. This implementation automatically generates a user-interface of the desired workflow for ease of use. The flexibility of the framework is not compromised by the UI frontend, since functionality is implemented in the Java backend. The implemented features of the DVP include import of cell morphologies, computation and visualization of 3D density maps, 1D density profiles, and cortical landmarks.

Density maps are useful in discriminating cell types and are an important tool for analysis and classification of neuronal morphologies (see Jiang et al., 2015). In particular 1D and 2D density maps have been proven useful in the past for cell morphology analysis. Here, a data set provided by the Feldmeyer group was analyzed using the DVP in order to demonstrate the functionality of the open-source toolbox. Using the visual output and the 1D density profiles computed by the DVP cell types fall into distinct clusters that can be used to classify cells based on the DVP output. The involved measurements neglect the detailed fine-grained neuromorphology and instead uses 3D density in full 3D space together with projections onto lower-dimensional subspaces to add insight into the interconnectivity patterns of specific cell types.

In contrast to this approach, morphometric statistics quantify the complex branching of neuronal structures, e.g., axons and dendrites interconnectivity, and generate summary statistics for the involved measures. From literature and recent analysis by Jiang et al. (2015) these two approaches yield the best result when classifying different cell categories. Sholl intersection analysis can be used as an intermediate approach between density maps and morphometric statistics. Since the presented DVP is fully open-source, additional methods, like morphometric statistics, or Sholl intersections analysis can be readily added to the framework once the data is imported which is already possible for supported data types in Table 1. In the presented examples (see Figure 6), the 1D density maps can be used to discriminate between the two different cell types.

Given the flexibility of VRL-Studio to integrate additional morphometric evaluators, the DVP framework can be further developed by the research community to couple graph theory metrics or neuroanatomical measurements. Since these types of cell type discrimination algorithms are not trivial in the sense of robustness, density maps are a robust addition to existing tools. The DVP framework was developed to offer a flexible platform to integrate heterogeneous classification tools and with an open-source policy in mind will ideally further promote collaborative research advances in automatic cell classification.

## Code

For the density visualization the data set provided in Emmenegger et al. (2018) was utilized. The code used for the density visualization and analysis can be found on Github in the following repository http://github.com/stephanmg/VRL-SWC-Density-Vis.

## Data Availability Statement

The raw data supporting the conclusions of this article will be made available by the authors, without undue reservation, to any qualified researcher.

## Author Contributions

SG implemented the density visualization pipeline. GQu analyzed the data. GQi provided the morphological data files and biological expertise. SG, GQu, and GQi wrote the manuscript.

## Conflict of Interest

The authors declare that the research was conducted in the absence of any commercial or financial relationships that could be construed as a potential conflict of interest.

## Footnotes

^1^http://alleninstitute.github.io/AllenSDK/

^2^http://vrl-studio.mihosoft.eu

^3^http://neuronland.org

^4^As by Wednesday 15th April, 2020.

^5^https://vrl-studio.mihosoft.eu/

^6^https://github.com/VRL-Studio/VRL

^7^https://vrl-studio.mihosoft.eu/

## References

[B1] Aguiar P. Sousa M. Szucs P. (2013). Versatile morphometric analysis and visualization of the three-dimensional structure of neurons. Neuroinformatics 11, 393–403. 10.1007/s12021-013-9188-z 23765606

[B2] Ascoli G. A. Donohue D. E. Halavi M. (2007). NeuroMorpho.org: a central resource for neuronal morphologies. J. Neurosci. 27, 9247–9251. 10.1523/JNEUROSCI.2055-07.2007 17728438 PMC6673130

[B3] Ascoli G. A. Krichmar J. Scorcioni R. Nasuto S. Senft S. (2001). Computer generation and quantitative morphometric analysis of virtual neurons. Anat. Embryol. (*Berl)*. 204, 283–301. 10.1007/s004290100201 11720234

[B4] Bota M. Swanson L. W. (2007). The neuron classification problem. Brain Res. Rev. 56, 79–88. 10.1016/j.brainresrev.2007.05.005 17582506 PMC2150566

[B5] Braitenberg V. Schüz A. (eds.). (1991). “Peters' rule and white's exceptions,” in Cortex: Statistics and Geometry of Neuronal Connectivity, 1st ed (Berlin-Heidelberg: Springer-Verlag), 109–112. 10.1007/978-3-662-02728-8_21

[B6] Breit M. Stepniewski M. Grein S. Gottmann P. Reinhardt L. Queisser G. (2016). Anatomically detailed and large-scale simulations studying synapse loss and synchrony using NeuroBox. Front. Neuroanat. 10:8. 10.3389/fnana.2016.00008 26903818 PMC4751272

[B7] Bria A. Iannello G. Onofri L. Peng H. (2016). Terafly: real-time three-dimensional visualization and annotation of terabytes of multidimensional volumetric images. Nat. Methods 13:192. 10.1038/nmeth.3767 26914202

[B8] Budd J. M. L. Cuntz H. Eglen S. J. Krieger P. (2015). Quantitative analysis of neuroanatomy. Front. Neuroanat. 9:143. 10.3389/fnana.2015.00143 26617494 PMC4641246

[B9] Cannon R. Turner D. Pyapali G. Wheal H. (1998). An on-line archive of reconstructed hippocampal neurons. J. Neurosci. Methods 84, 49–54. 10.1016/S0165-0270(98)00091-0 9821633

[B10] Costa L. d. F Rodrigues F. A. Travieso G. Villas Boas P. (2007). Characterization of complex networks: a survey of measurements. Adv. Phys. 56, 167–242. 10.1080/00018730601170527

[B11] Costa L. d. F Velte T. J. (1999). Automatic characterization and classification of ganglion cells from the salamander retina. J. Comp. Neurol. 404, 33–51. 10.1002/(SICI)1096-9861(19990201)404:1<33::AID-CNE3>3.0.CO;2-Y 9886023

[B12] Costa L. d. F (1995). Computer vision-based morphometric characterization of neural cells. Rev. Sci. Instr. 66, 3770–3773. 10.1063/1.1145435

[B13] Cuntz H. Forstner F. Borst A. Husser M. (2010). One rule to grow them all: a general theory of neuronal branching and its practical application. PLoS Comput. Biol. 6:e1000877. 10.1371/journal.pcbi.1000877 20700495 PMC2916857

[B14] Cuntz H. Forstner F. Haag J. Borst A. (2008). The morphological identity of insect dendrites. PLoS Comput. Biol. 4:e1000251. 10.1371/journal.pcbi.1000251 19112481 PMC2588660

[B15] Cuntz H. Mathy A. Häusser M. (2012). A scaling law derived from optimal dendritic wiring. Proc. Natl. Acad. Sci. U.S.A. 109, 11014–11018. 10.1073/pnas.1200430109 22715290 PMC3390826

[B16] DeFelipe J. Lopez-Cruz P. Benavides-Piccione R. C B Larranaga P.. (2013). New insights into the classification and nomenclature of cortical gabaergic interneurons. Nat. Rev. Neurosci. 14, 202–216. 10.1038/nrn3444 23385869 PMC3619199

[B17] Dercksen V. J. Hege H.-C. Oberlaender M. (2014). The Filament Editor: an interactive software environment for visualization, proof-editing and analysis of 3D neuron morphology. Neuroinformatics 12, 325–339. 10.1007/s12021-013-9213-2 24323305

[B18] Egger R. Narayanan R. T. Guest J. M. Bast A. Udvary D. Messore L. F. (2020). Cortical output is gated by horizontally projecting neurons in the deep layers. Neuron 105, 122–137. 10.1016/j.neuron.2019.10.011 31784285 PMC6953434

[B19] Emmenegger V. Qi G. Wang H. Feldmeyer D. (2018). Morphological and functional characterization of non-fast-spiking GABAergic interneurons in layer 4 microcircuitry of rat barrel cortex. Cereb. Cortex 28, 1439–1457. 10.1093/cercor/bhx352 29329401 PMC6093438

[B20] Gillette T. A. Ascoli G. A. (2015). Topological characterization of neuronal arbor morphology via sequence representation: I-motif analysis. BMC Bioinformatics 16:216. 10.1186/s12859-015-0605-1 26156313 PMC4496917

[B21] Gillette T. A. Grefenstette J. (2009). On comparing neuronal morphologies with the constrained tree-edit-distance. Neuroinformatics 7, 191–194. 10.1007/s12021-009-9053-2 19636974

[B22] Gouwens N. W. Sorensen S. A. Berg J. Lee C. Jarsky T. Ting J.. (2019). Classification of electrophysiological and morphological neuron types in the mouse visual cortex. Nat. Neurosci. 22, 1182–1195. 10.1038/s41593-019-0417-0 31209381 PMC8078853

[B23] Halavi M. Hamilton K. Parekh R. Ascoli G. (2012). Digital reconstructions of neuronal morphology: three decades of research trends. Front. Neurosci. 6, 49–55. 10.3389/fnins.2012.00049 22536169 PMC3332236

[B24] Helmstaedter C. Staiger J. F. Sakman B. Feldmeyer D. (2008). Efficient recruitment of layer 2/3 interneurons by layer 4 input in single columns of rat somatosensory cortex. J. Neurosci. 28, 8273–8284. 10.1523/JNEUROSCI.5701-07.2008 18701690 PMC6670569

[B25] Heumann H. Wittum G. (2009). The tree-edit-distance, a measure for quantifying neuronal morphology. Neuroinformatics 7, 179–190. 10.1007/s12021-009-9051-4 19475518

[B26] Hoffer M. Poliwoda C. Wittum G. (2013). Visual reflection library: a framework for declarative GUI programming on the Java platform. Comp. Vis. Sci. 16, 181–192. 10.1007/s00791-014-0230-y

[B27] Jefferis G. S. X. E. Potter C. J. Chan A. Marin E. C. Rohlfing T. Maurer C. R. J.. (2007). Comprehensive maps of Drosophila higher olfactory centers: spatially segregated fruit and pheromone representation. Cell 128, 1187–1203. 10.1016/j.cell.2007.01.040 17382886 PMC1885945

[B28] Jiang X. Shen S. Cadwell C. R. Berens P. Sinz F. Ecker A. S.. (2015). Principles of connectivity among morphologically defined cell types in adult neocortex. Science 350:6264. 10.1126/science.aac9462 26612957 PMC4809866

[B29] Kanari L. Dlotko P. Scolamiero M. Levi R. Shillcock J. Hess K.. (2018). A topological representation of branching neuronal morphologies. Neuroinformatics 16, 3–13. 10.1007/s12021-017-9341-1 28975511 PMC5797226

[B30] Kanari L. Ramaswamy S. Shi Y. Morand S. Meystre J. Perina R.. (2019). Objective morphological classification of neocortical pyramidal cells. Cereb. Cortex 23, 1719–1735. 10.1093/cercor/bhy339 30715238 PMC6418396

[B31] Koelbl C. Helmstaedter M. Lubke J. Feldmeyer D. (2015). A barrel-related interneuron in layer 4 of rat somatosensory cortex with a high intrabarrel connectivity. Cereb. Cortex 25, 713–725. 10.1093/cercor/bht263 24076498 PMC4318534

[B32] Levy R. B. Reyes A. D. (2012). Spatial profile of excitatory and inhibitory synaptic connectivity in mouse primary auditory cortex. J. Neurosci. 32, 5609–5619. 10.1523/JNEUROSCI.5158-11.2012 22514322 PMC3359703

[B33] Li Y. Wang D. Ascoli G. A. Mitra P. Wang Y. (2007). Metrics for comparing neuronal tree shapes based on persistent homology. PLoS ONE 12:e0182184. 10.1371/journal.pone.0182184 28809960 PMC5557505

[B34] Lorensen W. E. Cline H. E. (1987). Marching cubes: a high resolution 3D surface construction algorithm. SIGGRAPH Comput. Graph. 21, 163–169. 10.1145/37402.3742232149611

[B35] Lu Y. Trett K. Shain W. Carin L. Coifman R. R. Roysam B. (2013). “Quantitative profiling of microglia populations using harmonic co-clustering of arbor morphology measurements,” in 10th IEEE International Symposium on Biomedical Imaging: From Nano to Macro, ISBI 2013, 7–11 April, 2013 (San Francisco, CA: IEEE), 1360–1363. 10.1109/ISBI.2013.6556785

[B36] Lubke J. Roth A. Feldmeyer D. Sakmann B. (2003). Morphometric analysis of the columnar innervation domain of neurons connecting layer 4 and layer 2/3 of juvenile rat barrel cortex. Cereb. Cortex 13, 1051–1063. 10.1093/cercor/13.10.1051 12967922

[B37] Markram H. Muller E. Ramaswamy S. Reimann M. W. Abdellah M. Sanchez C. A.. (2015). Reconstruction and simulation of neocortical microcircuitry. Cell 163, 456–492. 10.1016/j.cell.2015.09.029 26451489

[B38] Narayanan R. T. Egger R. Johnson A. S. Mansvelder H. D. Sakmann B. de Kock C. P.. (2015). Beyond columnar organization: cell type- and target layer-specific principles of horizontal axon projection patterns in rat vibrissal cortex. Cereb. Cortex 25, 4450–4468. 10.1093/cercor/bhv053 25838038 PMC4816792

[B39] Oberlaender M. de Kock C. P. J. Bruno R. M. Ramirez A. Meyer H. S. Dercksen V. J.. (2011). Cell type-specific three-dimensional structure of thalamocortical circuits in a column of rat vibrissal cortex. Cereb. Cortex 22, 2375–2391. 10.1093/cercor/bhr317 22089425 PMC3432239

[B40] Packer A. M. McConnel D. J. Fino E. Yuste R. (2013). Axo-dendritic overlap and laminar projection can explain interneuron connectivity to pyramidal cells. Cereb. Cortex 23, 2790–2802. 10.1093/cercor/bhs210 22941716 PMC3968298

[B41] Peng H. Ruan Z. Long F. Simpson J. H. Myers E. W. (2010). V3D enables real-time 3D visualization and quantitative analysis of large-scale biological image data sets. Nat. Biotechnol. 28, 348–353. 10.1038/nbt.1612 20231818 PMC2857929

[B42] Peng H. Tang J. Xiao H. Bria A. Zhou J. Butler V.. (2014). Virtual finger boosts three-dimensional imaging and microsurgery as well as terabyte volume image visualization and analysis. Nat. Commun. 5:4342. 10.1038/ncomms5342 25014658 PMC4104457

[B43] Peters A. Feldman M. (1976). The projection of the lateral geniculate nucleus to area 17 of the rat cerebral cortex. J. Neurocytol. 5, 63–84. 10.1007/BF01176183 1249593

[B44] Peters A. Payne B. (1983). Numerical relationships between geniculocortical afferents and pyramidal cell modules in cat primary visual cortex. Cereb. Cortex 3, 69–78. 10.1093/cercor/3.1.69 8439740

[B45] Ramón y Cajal S. (1899). Textura del sistema nervioso del hombre y de los vertebrados. Madrid: Gobierno Aragon - Centro Libro.

[B46] Scala F. Kobak D. Shan S. Bernaerts Y. Laturnus S. Cadwell C. R. (2019). Layer 4 of mouse neocortex differs in cell types and circuit organization between sensory areas. Nat. Commun. 10:4174 10.1038/s41467-019-12769-3 31519874 PMC6744474

[B47] Scorcioni R. Polavaram S. Ascoli G. A. (2008). L-Measure: a web-accessible tool for the analysis, comparison and search of digital reconstructions of neuronal morphologies. Nat. Protoc. 3:866. 10.1038/nprot.2008.51 18451794 PMC4340709

[B48] Seung H. Sumbul U. (2014). Neuronal cell types and connectivity: lessons from the retina. Neuron 83, 1262–1272. 10.1016/j.neuron.2014.08.054 25233310 PMC4206525

[B49] Stepanyants A. Chklovskii D. B. (2005). Neurogeometry and potential synaptic connectivity. Trends Neurosci. 28, 387–394. 10.1016/j.tins.2005.05.006 15935485

[B50] Stepniewski M. Breit M. Hoffer M. Queisser G. (2019). NeuroBox: computational mathematics in multiscale neuroscience. Comput. Vis. Sci. 20, 111–124. 10.1007/s00791-019-00314-0 PMC7583561 33100898

[B51] Stockley E. W. Cole H. M. Brown A. D. Wheal H. V. (1993). A system for quantitative morphological measurement and electronic modelling of neurons: three-dimensional reconstruction. J. Neurosci. Methods 47, 39–51. 10.1016/0165-0270(93)90020-R 8321013

[B52] Sümbül U. Song S. McCulloch K. Becker M. Lin B. Sanes J. R.. (2014a). A genetic and computational approach to structurally classify neuronal types. Nat. Commun. 5:3512. 10.1038/ncomms4512 24662602 PMC4164236

[B53] Sümbül U. Zlateski A. Vishwanathan A. Masland R. H. Seung H. S. (2014b). Automated computation of arbor densities: a step toward identifying neuronal cell types. Front. Neuroanat. 8:139. 10.3389/fnana.2014.00139 25505389 PMC4243570

[B54] Sunkin S. M. Ng L. Lau C. Dolbeare T. Gilbert T. L. Thompson C. L.. (2012). Allen Brain Atlas: an integrated spatio-temporal portal for exploring the central nervous system. Nucleic Acids Res. 41, D996–D1008. 10.1093/nar/gks1042 23193282 PMC3531093

[B55] Uylings H. B. M. van Pelt J. (2002). Measures for quantifying dendritic arborizations. Netw. Comput. Neural Syst. 13, 397–414. 10.1088/0954-898X_13_3_309 12222821

[B56] Wolf S. Grein S. Queisser G. (2013). Employing NeuGen 2.0 to automatically generate realistic morphologies of hippocampal neurons and neural networks in 3D. Neuroinformatics 11, 137–148. 10.1007/s12021-012-9170-1 23111491

[B57] Zeng H. Sanes J. R. (2017). Neuronal cell-type classification: challenges, opportunities and the path forward. Nat. Rev. Neurosci. 18, 530–546. 10.1038/nrn.2017.85 28775344

