# Supplementary figures and images for: Density Visualization Pipeline: A Tool for Cellular and Network Density Visualization and Analysis
Source: Front Comput Neurosci. 2020 Jun 26;14:42. doi: 10.3389/fncom.2020.00042 (PMC7333680; doi:10.3389/fncom.2020.00042)

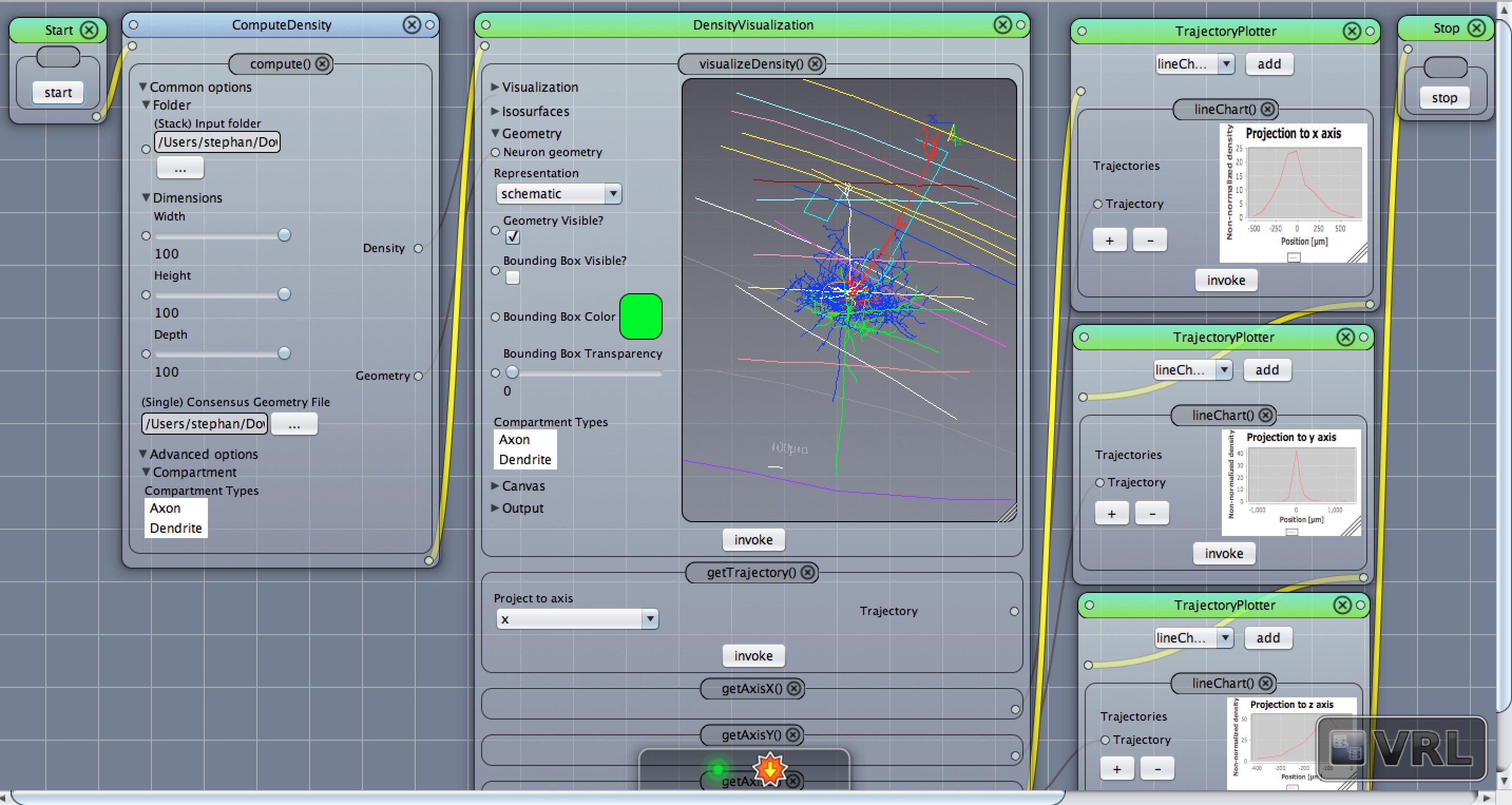

Supplement: Supplementary file 1 [file Image_1.jpeg]

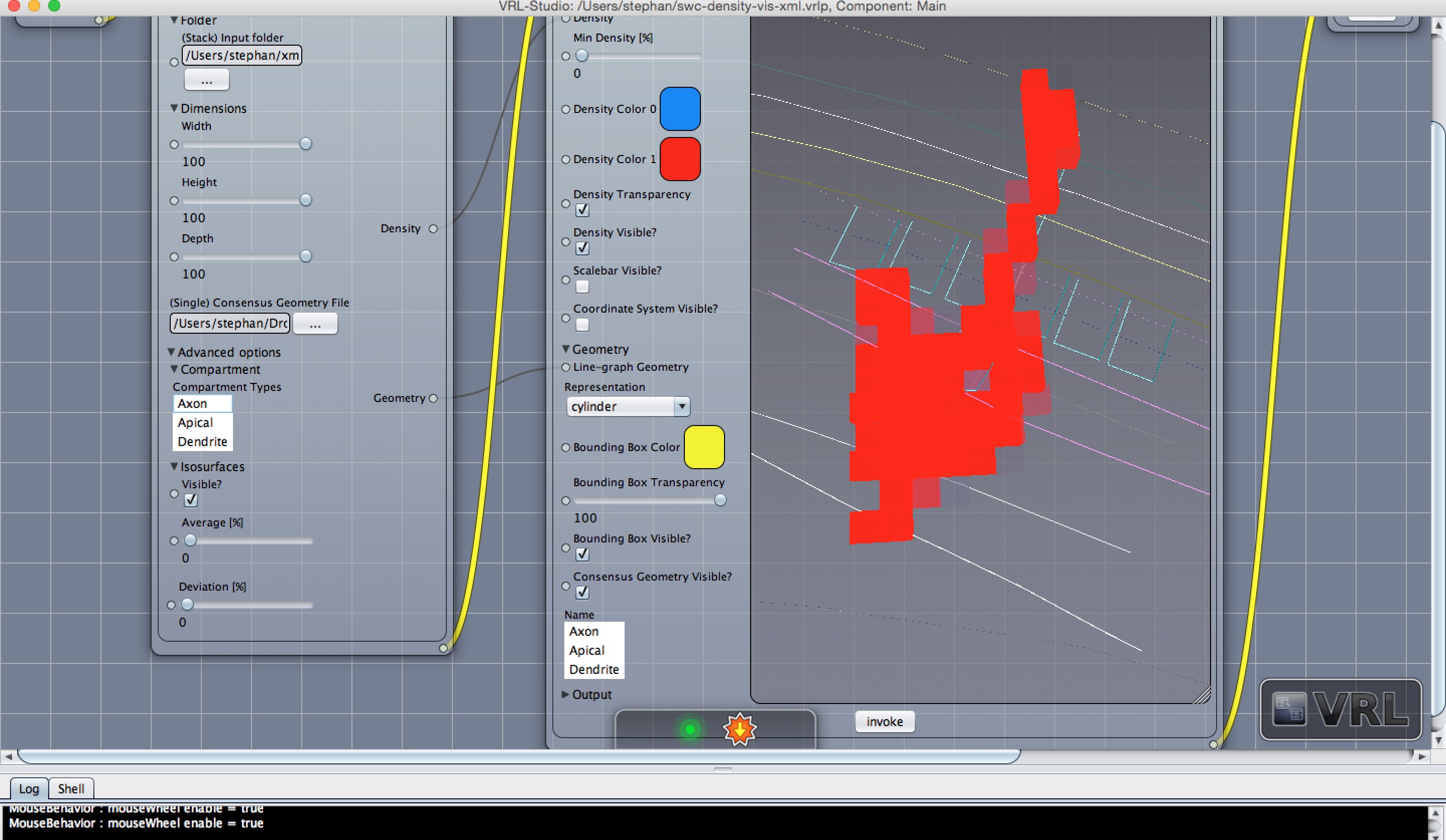

Supplement: Supplementary file 2 [file Image_2.jpeg]

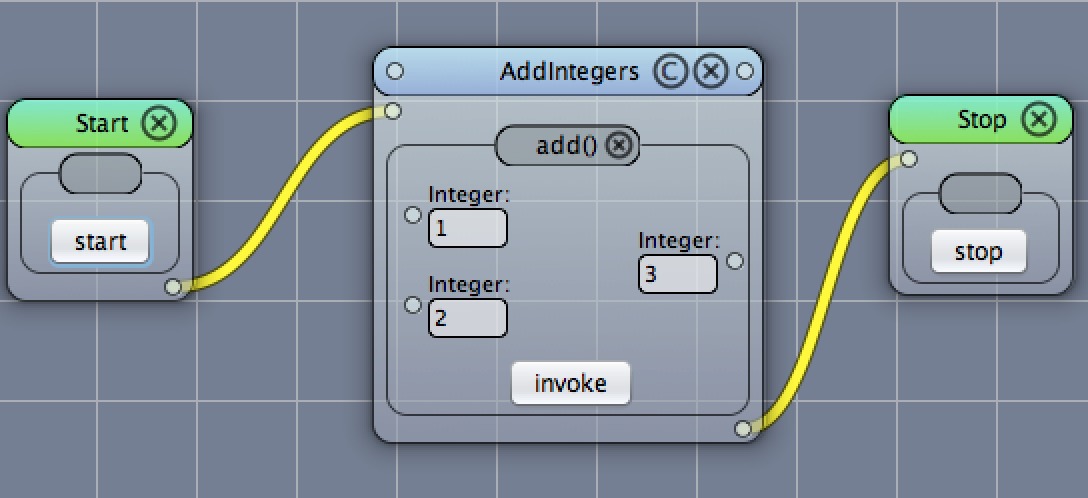

Supplement: Supplementary file 4 [file Image_4.JPEG]
