# Supplementary material for: Density Visualization Pipeline: A Tool for Cellular and Network Density Visualization and Analysis
Source: Front Comput Neurosci. 2020 Jun 26;14:42. doi: 10.3389/fncom.2020.00042 (PMC7333680; doi:10.3389/fncom.2020.00042)
Supplement: Supplementary file 5 [file Data_Sheet_1.pdf]

Supplementary Material: Density Visualization by visual programming

1 SUPPLEMENTARY DATA

Not applicable.

2 SUPPLEMENTARY TABLES AND FIGURES

2.1 Tables

Not applicable.

2.2 Figures

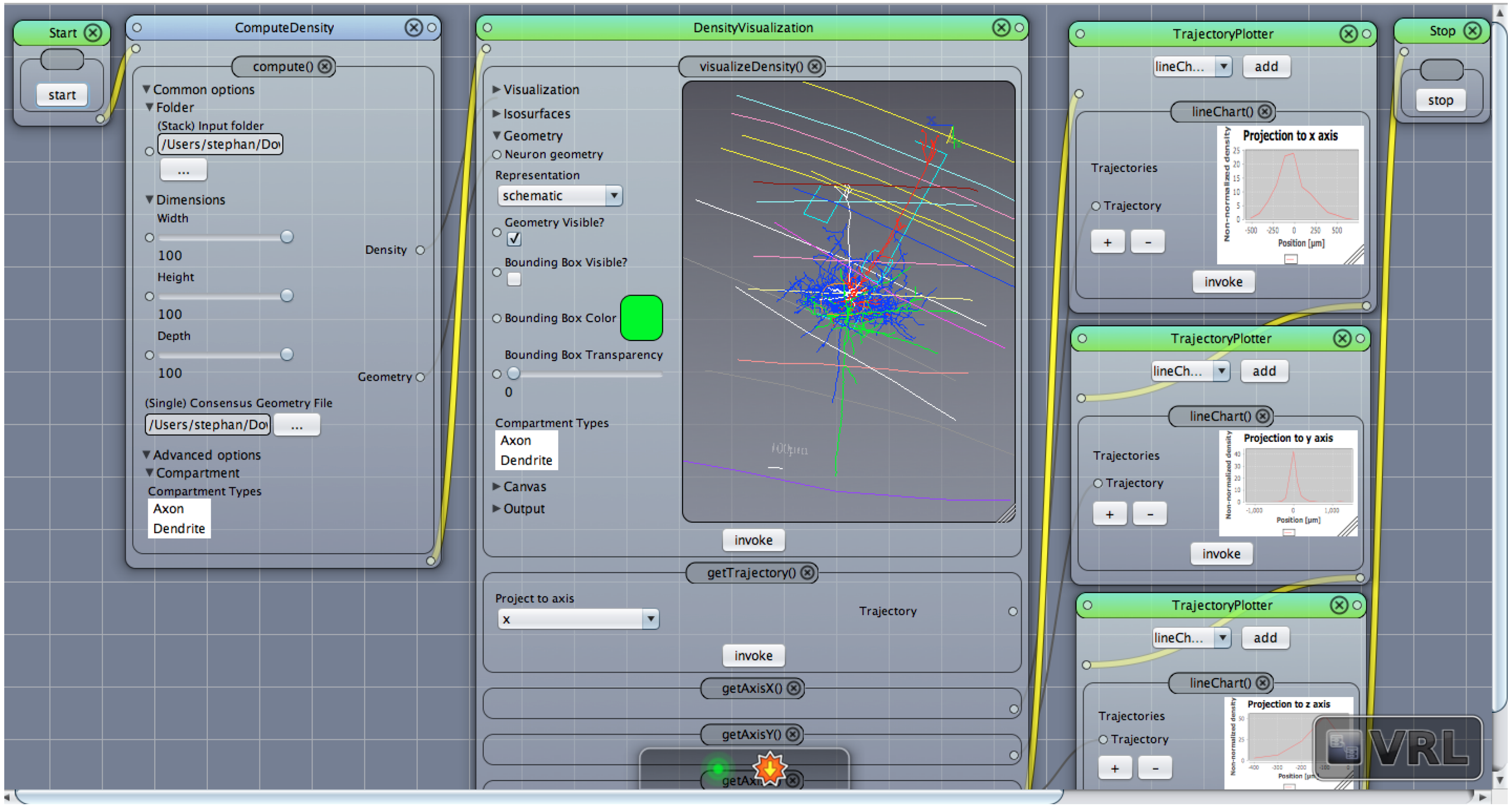

Figure S1. Schematic morphology. Displayed is the main Canvas view in VRL-Studio. Note that the voxel density view is not shown but instead a neuronal morphology which contains layer boundaries. Scale bar is visible in the bottom part of the geometry giving the user a hint of the size of the neuron of interest. Upper left part displays in addition a coordinates system and the bounding box of the neuron is omitted. The density data is pipelined into the three projectors which project the data to the x-, y- and z-axes.

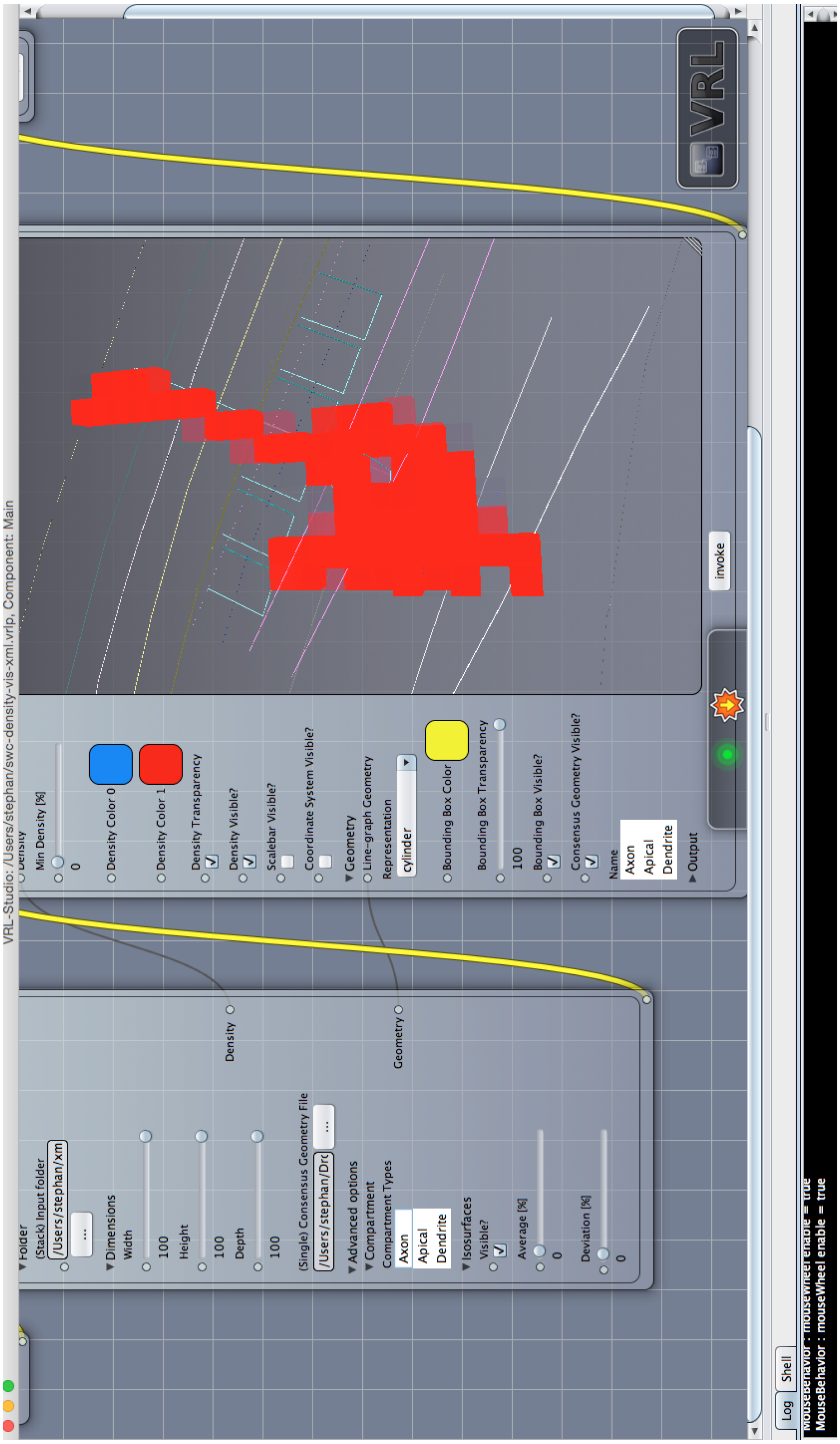

Figure S2. Density view with layer borders. Displayed is the main Canvas view in VRL-Studio. Layer boundaries are shown of a neuron of interest, density is visualized, however the geometry of the neuron is not considered.

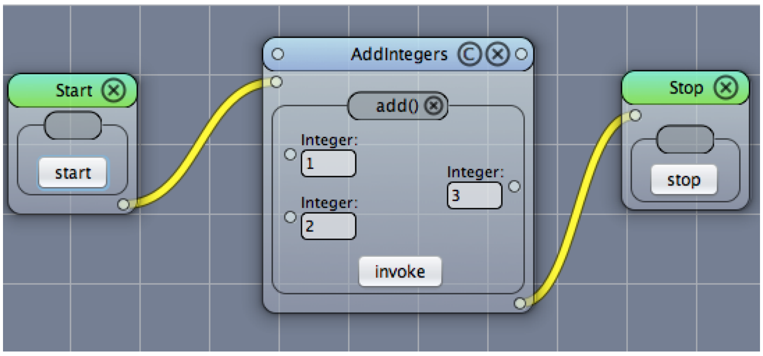

Figure S3. Graphical representation of the adding two numbers component. Two numbers can be inputed via the left-most two text input fields (Integer) to produce a third number on the right-most text input field (Integer). Note the possibility to feed in integers at the left-most circular markers and to output the integer into another component by means of the circular marker on the right-most side of the component. Notice the circled compile button on the top-right corner of the components (AddIntegers) title bar. This will allow the user by pressing to decompile the component and make the underlying source code visible.
